# Synthesis of positive plasmas with known chromosomal abnormalities for validation of non-invasive prenatal screening

**DOI:** 10.3389/fgene.2023.971087

**Published:** 2023-01-16

**Authors:** Zhongxia Qi, Jingwei Yu

**Affiliations:** Department of Laboratory Medicine, University of California, San Francisco, San Francisco, CA, United States

**Keywords:** non-invasive prenatal screening (NIPS), synthetic plasma, NIPS validation, cell free DNA, fetal fraction

## Abstract

Non-invasive prenatal screening (NIPS) is a DNA sequencing-based screening test for fetal aneuploidies and possibly other pathogenic genomic abnormalities, such as large deletions and duplications. Validation and quality assurance (QA) of this clinical test using plasmas with and without targeted chromosomal abnormalities from pregnant women as negative and positive controls are required. However, the positive plasma controls may not be available for many laboratories that are planning to establish NIPS. Limited synthetic positive plasmas are commercially available, but the types of abnormalities and the number/quantity of synthetic plasmas for each abnormality are insufficient to meet the minimal requirements for the initial validation. We report here a method of making synthetic positive plasmas by adding cell-free DNA (cfDNA) isolated from culture media of prenatal cells with chromosomal abnormalities to the plasmas from non-pregnant women. Thirty-eight positive plasmas with various chromosomal abnormalities, including autosomal and sex chromosomal aneuploidies, large deletions and duplications, were synthesized. The synthetic plasmas were characterized side-by-side with real positive plasmas from pregnant women and commercially available synthetic positive plasmas using the Illumina VeriSeq NIPT v2 system. All chromosomal abnormalities in the synthetic plasmas were correctly identified with the same testing sensitivity and specificity as in the real and commercial synthetic plasmas. The findings demonstrate that the synthetic positive plasmas are excellent alternatives of real positive plasmas for validation and QA of NIPS. The method described here is simple and straightforward, and can be readily used in clinical genetics laboratories with accessibility to prenatal cultures.

## 1 Introduction

The discovery of cell-free DNA (cfDNA) of fetal origin in blood plasma of pregnant women paved a new way for non-invasive prenatal screening (NIPS) (Lo et al., 1997). With advances in next-generation sequencing (NGS) technology, tens of millions of short sequence tags can be generated from cfDNA in a single maternal plasma sample. By counting the number of sequence tags mapped to each chromosome, fetal aneuploidies can be correctly detected. This accurate and reliable genomic screening for common fetal aneuploidies clearly outperforms the traditional serum protein screening (Chiu et al., 2008; Fan and Quake, 2010; Norton et al., 2015). NIPS has transformed prenatal care in countries and regions where it is available (Norton, 2022).

As a screening test, NIPS is routinely offered to women at as early as 10 weeks’ gestation. This test can be established in clinical genetics laboratories using commercially available platforms, for example the Illumina VeriSeq NIPT v2 system, or laboratory developed sequencing and bioinformatic pipelines. In either way, clinical validation and continuous monitoring of NIPS performance using both negative and positive plasma controls are required to ensure the test is performed appropriately. Negative plasmas can be obtained from female donors with normal pregnancies following appropriate protocols. However, positive plasmas that carry fetal cfDNA with targeted chromosomal abnormalities are usually very difficult to collect in a timely manner, in particular for laboratories new to this test, due to limited availability of such positive specimens. Although synthetic positive plasmas are commercially available, they are usually insufficient for the initial validation due to limited abnormality types and sample quantity. Therefore, development of reliable alternatives of the positive plasmas for NIPS validation and QA is needed to help and facilitate applications of NIPS. We describe here a simple method of making synthetic positive plasmas that are reliable and excellent alternatives of positive maternal plasmas for validation and monitoring NIPS performance.

## 2 Materials and equipment

### 2.1 Materials

Thirty-eight de-identified culture media were collected from backup cultures of chorionic villus cells or amniocytes that were submitted for prenatal diagnosis at the University of California San Francisco (UCSF) Clinical Cytogenetics Laboratory after reporting.

Twenty de-identified remaining plasmas of phenotypically normal non-pregnant females (age 20–42 years old) were collected after testing pathogens of infectious diseases at the UCSF Clinical Microbiology Laboratory. These samples that would be otherwise discarded were used as donor plasmas to make synthetic positive plasmas.

Two maternal blood samples from pregnancies with fetal aneuploidies were collected in Cell-Free DNA BCT tubes (Streck, Nebraska, United States) after obtaining the consent of each individual.

In addition, six synthetic positive plasmas, including two with trisomy 21, two with trisomy 18, and two with trisomy 13, were purchased from SeraCare Life Sciences (SeraCare Life Sciences, Massachusetts, United States).

Two hundred negative control plasmas with normal fetal cfDNA for NIPS system validation and training were provided by Illumina (Illumina, California, United States).

### 2.2 Reagents and kits

AmnioMAX-II complete media (ThermoFisher Scientific, Massachusetts, United States).

QIAamp MinElute ccfDNA kits (Qiagen, Hilden, Germany).

High sensitivity DNA kit (Agilent, California, United States).

VeriSeq NIPT Extraction and Library prep kit (Illumina, California, United States).

### 2.3 Equipment

Agilent 2100 Bioanalyzer (Agilent, California, United States).

Avanti J-15R centrifuge (Beckman Coulter, Indiana, United States).

Eppendorf MiniSpin plus centrifuge (Eppendorf, Connecticut, United States).

Corning 25 cm^2^ rectangular canted neck cell culture flask with vent cap (T25) (Corning, New York, United States).

Corning sterile 15 mL plastic conical centrifuge tube, graduated polypropylene, RNase & DNase-free (Corning, New York, United States).

Eppendorf 1.5 mL safe-lock clear tube (Eppendorf, Connecticut, United States).

Illumina VeriSeq NIPT v2 system (Illumina, California, United States).

Microlab STAR liquid handling system (Hamilton, Nevada, United States).

## 3 Methods

### 3.1 Isolation of plasma

Approximately 10 mL blood sample collected in a Cell-Free DNA BCT tube was centrifuged at 1,000 g for 10 min with centrifuge break off (Avanti J-15R centrifuge). The supernatant was then transferred to four 1.5 mL centrifuge tubes (1.1 mL plasma/tube).

Each tube with 1.1 mL plasma was further centrifuged at 5,600 g for 10 min (Eppendorf MiniSpin plus centrifuge), and 1.0 mL supernatant was transferred to a new centrifuge tube.

Isolated plasma could be stored at 4°C for up to 10 days. They could also be stored at −80°C for up to 2 years.

### 3.2 Extraction of cfDNA from culture media and from donor plasmas

Chorionic villus cells or amniocytes were first cultured to about 90% confluence in a T25 flask following a standard protocol (Segeritz and Vallier, 2017). The culture was then fed with 5 mL fresh AmnioMAX complete medium.

Three to 5 days after feeding (depending on cell growth), 3.0 mL culture medium was transferred from the flask into a 15 mL centrifuge tube and centrifuged at 1,000 g for 10 min with centrifuge break off (Avanti J-15R centrifuge).

Approximately 2.2 mL supernatant was transferred to two 1.5 mL centrifuge tubes (1.1 mL plasma/tube) (Eppendorf) and then centrifuged at 5,600 g for 10 min (Eppendorf MiniSpin plus centrifuge).

Two mL supernatant (1.0 mL from each tube) was used for cfDNA extraction. CfDNA was extracted using QIAamp MinElute ccfDNA Kit following the manufacture’s instruction. CfDNA was eluted into 25.0 µL nuclease-free water provided in the kit and was checked for fragment size and quantity on Bioanalyzer using Agilent high sensitivity DNA kit following the kit instruction.

CfDNA from six donor plasmas was also extracted and measured in the same way to estimate the average concentration of the background cfDNA in the donor plasmas.

### 3.3 Synthesis of positive plasmas

Approximately 1.0 ng short cfDNA (130–190 bp) with targeted chromosomal abnormalities from a culture medium was added to 1.0 mL normal female donor plasma collected through step 3.1 to make a synthetic positive plasma. The expected average fraction of the cfDNAs from culture media in the synthetic positive plasmas is approximately 7%.

### 3.4 Characterization of synthetic positive plasmas for detecting targeted abnormalities

The synthetic positive plasmas were characterized using the Illumina VeriSeq NIPT v2 system according to the manufacturer’s instruction. Briefly, cfDNA was extracted and the sequencing library was prepared using VeriSeq NIPT Extraction and Library Prep kits (Illumina) in Microlab STAR liquid handling system (Hamilton). The sample libraries were pooled and pair-end sequenced (36x2 cycles) on NextSeq550 (Illumina). The sequencing data were analyzed by VeriSeq NIPT software v2 (www.illumina.com/NIPTsoftware). This software aligned the sequencing reads to human reference genome GRCh37/hg19 and used a counting-based algorithm to generate the log-likelihood ratio (LLR) scores for chromosomes, as well as NCV_X and NCV_Y scores for sex classification. LLR thresholds for calling a sample high or low risk of specific chromosome abnormalities were internally validated. Data generated from fragment length and coverage analysis were used to estimate fetal fraction by the software.

### 3.5 NIPS data visualization

The LLRs of the synthetic positive plasmas with trisomy 21, trisomy 18, and trisomy 13, as well as the fetal fractions from the VeriSeq NIPT supplementary reports were plotted in RStudio (2021.09.2) using ggplot2 (3.3.6) for data visualization.

## 4 Results

**FIGURE 1:**
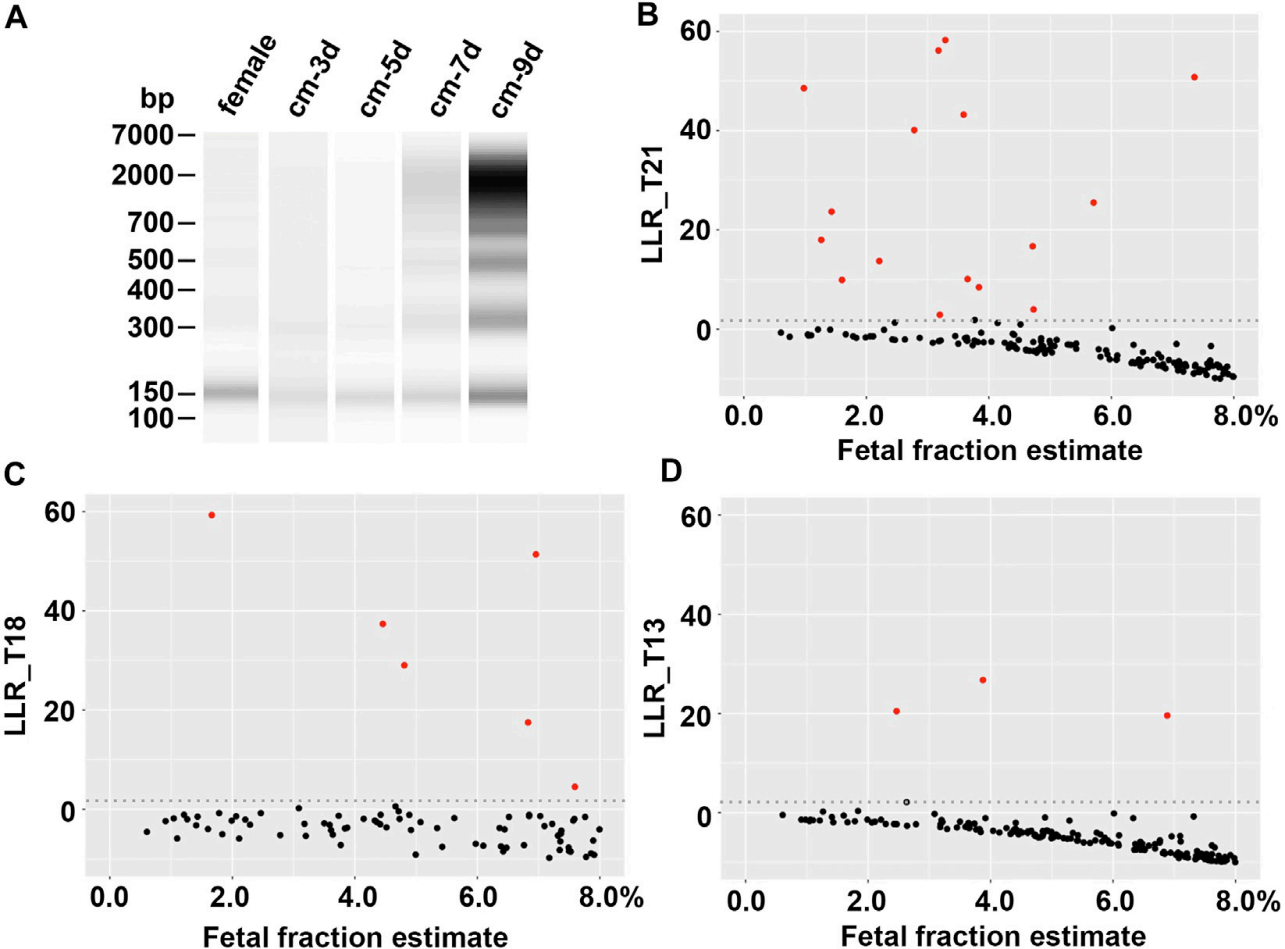
Evaluation of synthetic plasmas. **(A)** cfDNA size distribution. From left to right, cfDNA from a normal female plasma (female), culture media from a chorionic villus specimen with a 45,X karyotype collected at the day 3, 5, 7, and 9 of the culture, respectively (cm-3d, cm-5d, cm-7d, and cm-9d). **(B)**, **(C)**, and **(D)** Log likelihood ratios (LLRs) of synthetic plasmas (fetal fraction estimate ≤8%) with trisomy 21, trisomy 18, and trisomy 13, respectively. Red dot, synthetic positive plasma; black dot, negative maternal plasma; gray dotted line, LLR cutoff.

A total of 38 cfDNA samples with targeted chromosomal abnormalities were extracted from cell culture media of chorionic villus cells or amniocytes. The quantity and size of the cfDNA were determined on Bioanalyzer using Agilent high sensitivity DNA kit, which showed a size range from 100 bp to >1 kb in discontinuous clusters, including a major cluster of short sizes (130–190 bp) (Figure 1A). The average concentration of the cluster of short cfDNA is approximately 60 ng/mL in culture medium. This cluster of cfDNA was used to make synthetic plasmas, since its size range is most representable to the size range of fetal cfDNA in maternal plasmas (Kim et al., 2015; Jiang and Lo, 2016).

The average background cfDNA concentration measured by the same method in six donor plasmas was 13.7 ng/mL, ranging from 3.9 to 27.8 ng/mL. This range was in line with the findings of a broad survey of cfDNA from healthy donors (Raymond et al., 2017). Therefore, adding 1.0 ng abnormal cfDNA to 1.0 mL donor plasma resulted in an approximately 7% of average abnormal cfDNA fraction that would mimic the fetal fraction in the synthetic plasmas. This percentage was common in maternal plasmas based on the data reported in literatures. It was noteworthy that a wide range of fetal fraction (1%–15%) was estimated by the VeriSeq v2 system (Supplemental Table 1), most likely due to the various concentrations of the background cfDNA in the donor plasmas. In fact, this range of fetal fraction appeared to be consistent with a reported range (Canick et al., 2013; Artieri et al., 2017). We further analyzed the detectability of targeted chromosome abnormalities in synthetic positive plasmas with different fetal fractions to determine the sensitivity of the testing using the Illumina VeriSeq NIPT v2 system.

The abnormalities in the 38 synthetic positive plasmas included eighteen trisomy 21, six trisomy 18, four trisomy 13, four sex chromosomal aneuploidies (45,X and 47,XXY), one trisomy 7, one trisomy 16, two trisomy 20, one 10.5 Mb terminal deletion of chromosome 7p and 26.5 Mb terminal duplication of chromosome 9p, and one 26.3 Mb terminal duplication of chromosome 15q. All abnormalities in these synthetic positive plasmas were correctly detected by the Illumina VeriSeq NIPT v2 system (Supplementary Table S1). Figures 1B–D showed the LLRs of the synthetic plasmas with trisomy 21, trisomy 18, and trisomy 13, respectively, in comparison with that of the negative plasmas. Chromosomal abnormalities can be detected in the synthetic plasmas with the fetal fraction as low as 1% (Supplementary Table S1).

We also tested two real positive maternal plasmas with fetal trisomy 21 and trisomy 18, respectively, and six commercial positive plasmas, including two trisomy 21, two trisomy 18, and two trisomy 13 (SeraCare Life Sciences), in parallel with synthetic plasmas made in this study (Supplementary Table S1). There were no noticeable differences in sensitivity, specificity and other testing parameters between these samples and our synthetic plasmas.

## 5 Discussion

Short fetal cfDNAs in maternal plasmas were most likely derived from apoptosis (Jiang and Lo, 2016; Rostami et al., 2020). We noticed that cell culture media of prenatal specimens contain short cfDNA fragments that were probably derived from cell apoptosis during the culture. The sizes of such short cfDNA fragments are within the reported size range of fetal cfDNA in plasmas of pregnant women (Kim et al., 2015; Jiang and Lo, 2016). Therefore, it is possible to use this type of short cfDNA to make positive synthetic plasmas that could mimic maternal plasmas carrying fetal cfDNA with chromosomal abnormalities. Our study demonstrated that the synthetic positive plasmas can be readily and reliably used in clinical validation and QA of NIPS. The synthetic positive plasmas described in this study have been successfully used to validate and monitor the NIPS system in our laboratory, which are required by the national and state regulations. Negative synthetic plasma could also be synthesized using normal cfDNA as needed, although it may not be necessary since negative maternal plasmas are not difficult to collect.

Clinical laboratories that provide prenatal cytogenetic tests have unique advantages of making synthetic positive plasmas. It is required to maintain backup cultures for 2 weeks after reporting cytogenetic findings for all prenatal specimens in the United States. Other countries may also have similar requirements. Therefore, the laboratories can readily collect culture media of targeted abnormal cells from the backup cultures. The cfDNAs from the culture media can be directly used to make synthetic plasma after cfDNA extraction without further treatments. Synthetic positive plasmas may also be made using abnormal genomic DNA, but additional processes, such as fragmentation of long genomic DNA and isolation of short DNA, would be needed and those processes could be challenging.

The best time to collect short cfDNA from the culture medium of chorionic villus cell or amniocyte appears to be on day 3–5 after feeding the cells that grow at high confluency (∼90%) with fresh culture medium (Figure 1A). Short culture time might not be able to collect enough cfDNA; long culture time might result in more background of large DNA, probably due to increased cell death and reduced apoptotic activities.

De-identified remaining plasmas after pathogen testing from phenotypic normal non-pregnant females, which would be otherwise discarded, are readily to collect from clinical microbiology or immunology laboratories with appropriate protocols. It is less likely that a phenotypically normal non-pregnant female donor would carry aneuploidies that are usually associated with abnormal phenotypes. To ensure aneuploidy-free in the donors, each non-pregnant plasma was used to synthesize two positive plasmas with different abnormalities if possible. An abnormality of donor origin would be indicated if an abnormality showed in both synthetic plasmas.

While the synthetic plasmas can be used as controls on the Illumina VeriSeq NIPT v2 system, they have not been tested on other NIPS systems for validation of different methodologies, such as single nucleotide polymorphism (SNP)-based NIPS, cfDNA size selection, and targeted sequencing. We did not test cfDNA from culture media of other cell types. In addition, abnormal prenatal cell cultures may not be accessible for every laboratory in needs to make synthetic positive plasmas.

In conclusion, we reported a practical strategy of making synthetic positive plasmas that could be used for NIPS validation and QA. This method could be especially helpful for clinical genetics laboratories that plan to implement NIPS testing.

## Data Availability

The raw data supporting the conclusion of this article will be made available by the authors, without undue reservation.
