# Supplementary material for: Synthesis of positive plasmas with known chromosomal abnormalities for validation of non-invasive prenatal screening
Source: Front Genet. 2023 Jan 16;14:971087. doi: 10.3389/fgene.2023.971087 (PMC9886268; doi:10.3389/fgene.2023.971087)
Supplement: Supplementary file 1 [file DataSheet1.PDF]

Supplementary Table 1. Chromosome abnormalities detected in synthetic, maternal, and commercial plasmas.

Sample_Type	Sample_Name	Known Abnormality	Pregnancy Type	Screen Type	Flowcell	Predicted Sex	Anomaly Description	qc_flag	qc_reason	Fetal Fraction
Synthetic plasma	SYN_T21_1	47,XY,+21	Singleton	genomewide	H2JWLBGXJ	XY	DETECTED: +21	PASS	NONE	10%
Synthetic plasma	SYN_T21_2	47,XX,+21	Singleton	genomewide	H2JWLBGXJ	XX	DETECTED: +21	PASS	NONE	6%
Synthetic plasma	SYN_T21_3	47,XY,+21	Singleton	genomewide	H2JGFBGXJ	XY	DETECTED: +21	PASS	NONE	1%
Synthetic plasma	SYN_T21_4	47,XY,+21	Singleton	genomewide	H2JGFBGXJ	XY	DETECTED: +21	PASS	NONE	11%
Synthetic plasma	SYN_T21_5	47,XX,+21	Singleton	genomewide	H2JGFBGXJ	XX	DETECTED: +21	PASS	NONE	9%
Synthetic plasma	SYN_T21_6	47,XY,+21	Singleton	genomewide	H2JGFBGXJ	XY	DETECTED: +21	PASS	NONE	10%
Synthetic plasma	SYN_T21_7	47,XX,+21	Singleton	genomewide	H2JGFBGXJ	XX	DETECTED: +21	PASS	NONE	8%
Synthetic plasma	SYN_T21_8	47,XX,+21	Singleton	genomewide	H2JGFBGXJ	XX	DETECTED: +21	PASS	NONE	15%
Synthetic plasma	SYN_T21_9	47,XY,+21	Singleton	genomewide	H2JGFBGXJ	XY	DETECTED: +21	PASS	NONE	6%
Synthetic plasma	SYN_T21_10	47,XX,+21	Singleton	genomewide	H2KVNBGXJ	XX	DETECTED: +21	PASS	NONE	10%
Synthetic plasma	SYN_T21_11	47,XY,+21	Singleton	genomewide	H2KVNBGXJ	XY	DETECTED: +21	PASS	NONE	7%
Synthetic plasma	SYN_T21_12	47,XY,+21	Singleton	genomewide	H2KVNBGXJ	XY	DETECTED: +21	PASS	NONE	14%
Synthetic plasma	SYN_T21_13	47,XY,+21	Singleton	genomewide	H2KW7BGXJ	XY	DETECTED: +21	PASS	NONE	2%
Synthetic plasma	SYN_T21_14	47,XY,+21	Singleton	genomewide	H2KW7BGXJ	XY	DETECTED: +21	PASS	NONE	6%
Synthetic plasma	SYN_T21_15	47,XY,+21	Singleton	genomewide	H2NLTBGXJ	XY	DETECTED: +21	PASS	NONE	5%
Synthetic plasma	SYN_T21_16	47,XX,+21	Singleton	genomewide	H2T3VBGXJ	XX	DETECTED: +21	PASS	NONE	3%
Synthetic plasma	SYN_T21_17	47,XX,+21	Singleton	genomewide	H2T3VBGXJ	XX	DETECTED: +21	PASS	NONE	4%
Synthetic plasma	SYN_T21_18	47,XY,+21	Singleton	genomewide	HWG7CBGXJ	XY	DETECTED: +21	PASS	NONE	6%
Synthetic plasma	SYN_T18_1	47,XY,+18	Singleton	genomewide	H2JGFBGXJ	XY	DETECTED: +18	PASS	NONE	8%
Synthetic plasma	SYN_T18_2	47,XX,+18	Singleton	genomewide	H2JGFBGXJ	XX	DETECTED: +18	PASS	NONE	4%
Synthetic plasma	SYN_T18_3	47,XY,+18	Singleton	genomewide	H2KVNBGXJ	XY	DETECTED: +18	PASS	NONE	7%
Synthetic plasma	SYN_T18_4	47,XX,+18	Singleton	genomewide	H2NLTBGXJ	XX	DETECTED: +18	PASS	NONE	12%
Synthetic plasma	SYN_T18_5	47,XY,+18	Singleton	genomewide	H2T3TBGXJ	XY	DETECTED: +18	PASS	NONE	10%
Synthetic plasma	SYN_T18_6	47,XX,+18	Singleton	genomewide	H2T3TBGXJ	XX	DETECTED: +18	PASS	NONE	5%
Synthetic plasma	SYN_T13_1	47,XY,+13	Singleton	genomewide	H2T3NBGXJ	XY	DETECTED: +13	PASS	NONE	10%
Synthetic plasma	SYN_T13_2	47,XX,+13	Singleton	genomewide	H2T3NBGXJ	XX	DETECTED: +13	PASS	NONE	3%
Synthetic plasma	SYN_T13_3	47,XX,+13	Singleton	genomewide	H2T3NBGXJ	XX	DETECTED: +13	PASS	NONE	4%

Sample_Type	Sample_Name	Known Abnormality	Pregnancy Type	Screen Type	Flowcell	Predicted Sex	Anomaly Description	qc_flag	qc_reason	Fetal Fraction
Synthetic plasma	SYN_T13_4	47,XX,+13	Singleton	genomewide	HWJNMBGXJ	XX	DETECTED: +13	PASS	NONE	6%
Synthetic plasma	SYN_SEX_1	47,XXY	Singleton	genomewide	H2JGFBGXJ	XXY	DETECTED: XXY	PASS	NONE	5%
Synthetic plasma	SYN_SEX_2	45,X	Singleton	genomewide	H2KVNBGXJ	XO	DETECTED: XO	PASS	NONE	4%
Synthetic plasma	SYN_SEX_3	45,X	Singleton	genomewide	H2NLTBGXJ	XO	DETECTED: XO	PASS	NONE	3%
Synthetic plasma	SYN_SEX_4	45,X	Singleton	genomewide	H2T3VBGXJ	XO	DETECTED: XO	PASS	NONE	7%
Synthetic plasma	SYN_T7_1	47,XY,+7	Singleton	genomewide	H2KW7BGXJ	XY	DETECTED: +7	PASS	NONE	5%
Synthetic plasma	SYN_T16_1	47,XX,+16	Singleton	genomewide	H2KVNBGXJ	XX	DETECTED: +16	PASS	NONE	2%
Synthetic plasma	SYN_T20_1	47,XY,+20	Singleton	genomewide	H2T3TBGXJ	XY	DETECTED: +20	PASS	NONE	6%
Synthetic plasma	SYN_T20_2	47,XX,+20	Singleton	genomewide	HWGF2BGXJ	XX	DETECTED: +20	PASS	NONE	5%
Synthetic plasma	SYN_DELDUP_1	46,XX,der(7)t(7;9)(q36;p21): 10.5 Mb deletion within 7q36.1q36.3, 26.5 Mb duplication within 9p24.3p21.2 by microarray	Singleton	genomewide	H2KVNBGXJ	XX	DETECTED: del(7)(q36.1q36.3); dup(9)(p24.3p21.2)	PASS	NONE	7%
Synthetic plasma	SYN_DELDUP_2	46,XX,der(9)t(9;15)(q34.3;q24.2): 26.3 Mb duplication within 15q24.3q26.3 by microarray	Singleton	genomewide	H2T3TBGXJ	XX	DETECTED: dup(15)(q24.3q26.3)	PASS	NONE	6%
Positive maternal plasma	NIPSPOS_T21_FEMALE	High risk for trisomy 21 by NIPS	Singleton	genomewide	H2JGFBGXJ	XX	DETECTED: +21	PASS	NONE	17%
Positive maternal plasma	NIPSPOS_T18_FEMALE	High risk for trisomy 18 by NIPS	Singleton	genomewide	H2KVNBGXJ	XX	DETECTED: +18	PASS	NONE	10%
Commercial plasma from SeraCare	SERACARE_T21_MALE	Female witih trysomy 21	Singleton	genomewide	H2JWLBGXJ	XX	DETECTED: +21	PASS	NONE	9%
Commercial plasma from SeraCare	SERACARE_T21_FEMALE	Male witih trysomy 21	Singleton	genomewide	H2KVNBGXJ	XY	DETECTED: +21	PASS	NONE	10%
Commercial plasma from SeraCare	SERACARE_T18_MALE	Male witih trysomy 18	Singleton	genomewide	H2KVNBGXJ	XY	DETECTED: +18	PASS	NONE	7%
Commercial plasma from SeraCare	SERACARE_T18_FEMALE	Female witih trysomy 18	Singleton	genomewide	H2KW7BGXJ	XX	DETECTED: +18	PASS	NONE	11%
Commercial plasma from SeraCare	SERACARE_T13_MALE_1	Male witih trysomy 13	Singleton	genomewide	H2JWLBGXJ	XY	DETECTED: +13	PASS	NONE	15%
Commercial plasma from SeraCare	SERACARE_T13_MALE_2	Male witih trysomy 13	Singleton	genomewide	H2JWLBGXJ	XY	DETECTED: +13	PASS	NONE	14%
